# Cyberbullying detection: advanced preprocessing techniques & deep learning architecture for Roman Urdu data

**DOI:** 10.1186/s40537-021-00550-7

**Published:** 2021-12-22

**Authors:** Amirita Dewani, Mohsin Ali Memon, Sania Bhatti

**Affiliations:** Institute of Information and Communication Technologies, Department of Software Engineering, Mehran University of Engineering & Technology, Jamshoro, Sindh Pakistan

**Keywords:** Advanced preprocessing, Big data, Deep learning, Hate speech detection, Cyberbullying

## Abstract

Social media have become a very viable medium for communication, collaboration, exchange of information, knowledge, and ideas. However, due to anonymity preservation, the incidents of hate speech and cyberbullying have been diversified across the globe. This intimidating problem has recently sought the attention of researchers and scholars worldwide and studies have been undertaken to formulate solution strategies for automatic detection of cyberaggression and hate speech, varying from machine learning models with vast features to more complex deep neural network models and different SN platforms. However, the existing research is directed towards mature languages and highlights a huge gap in newly embraced resource poor languages. One such language that has been recently adopted worldwide and more specifically by south Asian countries for communication on social media is Roman Urdu i-e Urdu language written using Roman scripting. To address this research gap, we have performed extensive preprocessing on Roman Urdu microtext. This typically involves formation of Roman Urdu slang- phrase dictionary and mapping slangs after tokenization. We have also eliminated cyberbullying domain specific stop words for dimensionality reduction of corpus. The unstructured data were further processed to handle encoded text formats and metadata/non-linguistic features. Furthermore, we performed extensive experiments by implementing RNN-LSTM, RNN-BiLSTM and CNN models varying epochs executions, model layers and tuning hyperparameters to analyze and uncover cyberbullying textual patterns in Roman Urdu. The efficiency and performance of models were evaluated using different metrics to present the comparative analysis. Results highlight that RNN-LSTM and RNN-BiLSTM performed best and achieved validation accuracy of 85.5 and 85% whereas F1 score was 0.7 and 0.67 respectively over aggression class.

## Introduction

Cyberbullying (aka hate speech, cyberaggression and toxic speech) is a critical social problem plaguing today’s Internet users typically youth and lead to severe consequences like low self-esteem, anxiety, depression, hopelessness and in some cases causes lack of motivation to be alive, ultimately resulting in death of a victim [1]. Cyberbullying incidents can occur via various modalities. For example, it can take the form of sharing/ posting offensive video content or uploading violent images or sharing the pictures without permission of the owner etc. However, cyberbullying via textual content is far more common [2]. In Pakistan, the usage of internet, smartphones and social media has increasingly become prevalent these days and the very frequent users are youngsters. According to a report, more than 65% of all the users lie between 18 and 29, and typically women are more susceptible and unprotected. People often use offensive language, use hate speech, and become aggressive to bully celebrities, leaders, women and an individual [3]. In Pakistan, victims have reported life disturbing and annoying experiences and most of the victims are educated youngsters (age group of 21–30 years) [4]. The traffic in cyberspace has escalated significantly during covid-19 pandemic. A report “COVID 19 and Cyber Harassment”, released by DRF in 2020 highlights a great rise in the number of cyberbullying and harassment cases during the pandemic. The complaints registered with DRF’s Cyber Harassment Helpline were surged by 189% [5].

Recently, Roman Urdu language has been a contemporary trend and a viable language for communication on different social networking platforms. Urdu is national and official language of Pakistan and predominant among most communities across different regions. A survey statistic in [6] affirms that 300 million people are speaking Urdu language and approximately 11 million speakers are in Pakistan from which maximum users switched to Roman Urdu language for the textual communication, typically on social media. It is linguistically rich and morphologically complex language [7]. Roman Urdu language is highly variant with respect to word structures, writing styles, irregularities, and grammatical compositions. It is deficit of standard lexicon and available resources and hence become extremely challenging when performing NLP tasks.

An elaboration of script of Urdu instances and Roman Urdu is given in Table 1. Instances highlighted are describing anti-social behavior.

**Table 1:** Example script of Urdu and Roman Urdu instances

Urdu Language [RTL Script, Perso Arabic/Nastaliq]	Roman Urdu Language (LTR script, Romanian/Latin)
آپ نے عید کی پکس پوسٹ کیون نہیں کی	Aap ne edi ki pics post kiyon nahen ki
رنگ بـلکل کالا ہے تمہارا	Rang bahut kaala hai tumhara
بۂھت خوب ادا	Bahut khoob ada
اب جی سکو تو جی لو ورنا مرنا بہتر ہے	Ab jee sako to jee lo warna marna behtar hai

This paper addresses toxicity/cyberbullying detection problem in Roman Urdu language using deep learning techniques and advanced preprocessing methods including usage of lexicons/resource that are typically developed to accomplish this work. Intricacies in analyzing the structure and patterns behind these typical aggressive behaviors, typically in a newly adopted language, and forming it as a comprehensive computational task is very complicated. The major contributions of this study are formation of a slang and contraction mapping procedure along with slang lexicon for Roman Urdu language and development of hybrid deep neural network models to capture complex aggression and bullying patterns.

The rest of the paper is organized as follows: Review of existing literature is presented in "Related Work" Section. "Problem statement" Section states research gap and gives formal definition of the addressed problem. "Methodology" Section describes the steps of research methodology and techniques and models used for the experimentations. Advanced preprocessing steps applied on Roman Urdu data are elaborated in "Data Preprocessing on Roman Urdu microtext" section. Implementation of proposed model architecture and hyperparameter settings are discussed in "Experimental Setup" section. "Results and Discussion" Section highlights and discusses study results and finally "Conlusion" Section concludes the research work and provides future research directions.

## Related work

Due to the accretion of social media communication and adverse effects arising from its darker side on users, the field of automatic cyberbullying detection has become an emerging and evolving research trend [8]. Research work in [9] presents cyberbullying detection algorithm for textual data in English language. It is considered as one of the pioneers and highly cited research. They divided the task in text-classification sub problems related to sensitive topics and collected 4500 textual comments on controversial YouTube videos. This study implemented Naive Bayes, SVM and J48 binary and multiclass classifiers using general and specific feature sets. Study contributed in [10] applied deep learning architectures on Kaggle dataset and conducted experimental analysis to determine the effectiveness and performance of deep learning algorithms LSTM, BiLSTM, RNN and GRU in detecting antisocial behavior. Authors in [11] extracted data from four platforms i-e Twitter, YouTube, Wikipedia, and Reddit for developing an online hate classifier in English language using different classification techniques. Research carried out in [12] developed an automated approach to detect toxicity and unethical behavior in online communication using word embeddings and varying neural network layers. They suggested that LSTM layers and mimicked word embedding can uncover such behavior with good accuracy level.

Few of the studies in recent years has been contributed by researchers on other languages apart from English. Research work in [13] is unique and has gathered textual data from Instagram and twitter in Turkish language. They have implemented Naïve Bayes Multinomial, SVM, KNN and decision trees for cyberbullying detection along with Chi-square and information gain (IG) for feature selection. Work accomplished in [14] also addresses the problem of cyberaggression in Turkish language. The work extends comparison of different machine learning algorithms and found optimal results using Light Gradient Boosting Model. Van Hee, Cynthia, et al. in [15] proposed cyberbullying detection scheme for Dutch language. This is the first study on Dutch social media. Data was collected from ASKfm where users can ask and answer questions. The research uses default parameter settings for un-optimized linear kernel SVM based on n-grams and keyword system to identify bullying traces. F1 score for Dutch language was 61%. Problem of Arabic language cyberbullying detection was addressed and accomplished in [16]. This study used Dataiku DSS and WEKA for ML tasks. The data was scrapped from facebook and twitter. The study concluded that even though the detection approach was not comparable with the other studies in English language but overall Naive Bayes and SVM yield reasonable performance. Research work in [17] by Gomez-Adorno, Helena, et al. proposed automatic aggression detection for Spanish tweets. Several types of n-grams and linguistically motivated patterns were used but the best run could only achieve F1 score of 42.85%. Studies presented in [18–20] are based on automatic detection of cyberbullying content in German language. Research conducted in [18] proposed an approach based on SVM, CNN and ensemble model using unigram, bigrams and character N-grams for categorizing offensive tweets in German language. Research presented in [21] attempted for the very first time to identify bullying traces in Indonesian language. Association Rule mining and FP growth text mining were used to identify trends for bullying patterns in Jakarta and Surabaya cities using social media text. This baseline study on Indonesian language was further extended by Nurrahmi, Hani et al. in [22]. Study in [23] made first attempt to develop a corpus of code-mixed data considering Hindi and English language. They proposed a scheme for hate speech detection using N-grams and lexical features. An ensemble approach by combining the predictions of Convolutional Neural Network (CNN) and SVM algorithms were used for identifying such patterns. The weighted F1 score for Hindi language ranged between 0.37 and 0.55 for different experiments [24]. In the year 2019, Association for computational linguistics initiated the project for automatic detection of cyberbullying in Polish language [25]. Research conducted in [26] attempted to uncover cyberbullying patterns in Bengali language implementing passive aggressive, SVM and logistic regression. The optimum accuracy achieved was 78.1%. Recently, work contributed in [27] presented first study in Roman Urdu using lexicon based approach. The dataset was highly skewed comprising of only 2.2% toxic data. As according to [28], biased sampling and measurement errors are highly prone to classification errors when working on such datasets. Moreover, pattern detection based on predefined bullying and non-bullying lexicons were shortcomings of this study.

For automated detection of complex cyberbullying patterns, studies contributed by different scholars employ supervised, unsupervised, hybrid and deep learning models, vast feature engineering techniques, corpora, and social media platforms. However, the existing literature is mainly oriented towards unstructured data in English language. Some recent studies and projects have been initiated in other languages as discussed previously. To the best of our knowledge and literature review, no detailed work has been contributed in Roman Urdu to systematically analyze cyberbullying detection phenomenon using advanced preprocessing techniques (involving the usage of Roman Urdu resources) and deep learning approaches under different configurations.

## Problem statement

The escalated usage of social networking sites and freedom of speech has given optimal ground to individuals across all demographics for cyberbullying and cyberaggression. This leaves drastic and noticeable impacts on behavior of a victim, ranging from disturbance in emotional wellbeing and isolation from society to more severe and deadly consequences [29]. Automatic Cyberbullying detection has remained very challenging task since social media content is in natural language and is usually posted in unstructured free-text form leaving behind the language norms, rules, and standards. Evidently, there exists a substantial number of research studies which primarily focus on discovering cyberbullying textual patterns over diverse social media platforms as discussed previously in literature review section. However, most of the detection schemes and automated approaches formulated are for resource-rich and mature languages spoken worldwide. Roman Urdu is typically spoken in South Asia and is a highly resource deficient language. Hence this research puts novel efforts to propose data pre-processing techniques on Roman Urdu scripting and develop deep learning-based hybrid models for automated cyberbullying detection in Roman Urdu language. The outcomes of this study, if implemented, will assist cybercrime centers and investigation agencies for monitoring social media contents and in making cyberspace secure and safer place for all segments of society.

## Methodology

**Fig. 1:**
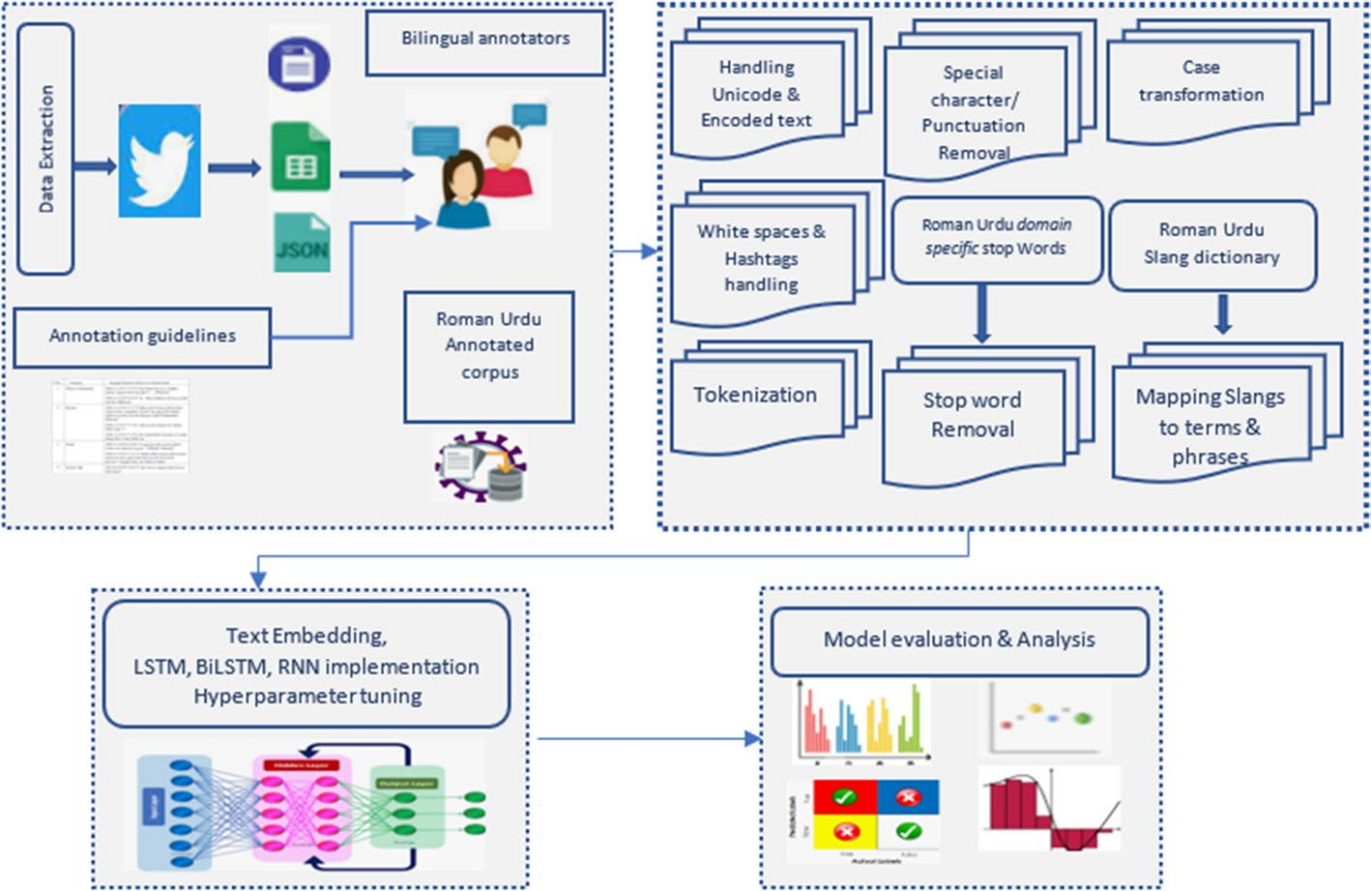
Proposed research methodology

The research methodology is depicted in Fig. 1.

The development of hate speech/cyberbullying corpus with minor skew and automated development of domain specific roman Urdu stop words is published in our previous work [30]. The work details formation of computational linguist resources. Further steps of methodology are discussed in subsequent sections. The Deep Neural Network (DNN) based techniques and models used for the experimentations are detailed below.

### Model description

#### Recurrent neural networks (RNN)

RNN [31] has been applied in literature for successive time series applications with temporal dependencies. An unfolded RNN can handle processing of current data by utilizing past data. Meanwhile, RNN has the issue of training long-term dependencies. This has been addressed by one of the RNN variant.

#### Long short-term memory networks (LSTM)

LSTM has been employed as an advanced version of RNN network. It resolves the shortcoming of RNN by applying memory cells also known as hidden layer units. Memory cells are controlled through three gates named as: input gate, output gate and forget gate. They have the self-connections which store the temporal state of network [31]. Input and output gates address and control the flow of information from memory cell input and output to rest of the network. The forget gate, usually called as a remember vector, transfers the information with higher weights from previous neuron to the next neuron. The forget gate is added to the memory cell. The information resides in memory depending upon the high activation results; the information will be stored in memory cell iff the input unit has high activation. However, the information will be transferred to next neuron if the output unit has high activation. Otherwise, input information with high weights resides in memory cell [31].

Mathematically, LSTM network can be described as [32]:

1 $$h_{t} = \, f \, \left( {W_{h} \cdot \, x_{t} + \, U_{h} \cdot \, h_{t - 1} + \, b_{h} } \right)$$

where *W*_*h*_* ∈ R*^*m*×*d*^ and *U*_*h*_* ∈ R*^*m*×*m*^ indicates weight matrices, *x*_*t*_ denotes the current word embedding, *b*_*h*_* ∈ R*^m^ refers to bias term, whereas *f(x*) is a non-linear function.

LSTM has more complex architecture including hidden states and tends to remember information for either short or long term. The hidden state [33] of LSTM is computed as follows:

2 $$f_{t} = \sigma (W_{f} \cdot \, x_{t} + \, U_{f} . \, h_{t - 1} + \, b_{f} )$$

$$i_{t} = \sigma (W_{i} \cdot \, x_{t} + \, U_{i} . \, h_{t - 1} + \, b_{i} )$$

$$o_{t} = \sigma (W_{o} \cdot \, x_{t} + \, U_{o} . \, h_{t - 1} + \, b_{o} )$$

$$c_{t} = f_{t} \circ c_{t} - {1 } + i_{t} \circ {\text{tanh }}(W_{c} \cdot \, x_{t} + \, U_{c} \cdot \, h_{t - 1} + \, b_{c} )$$

$$h_{t} = o_{t} \circ {\text{tanh }}\left( {c_{t} } \right)$$

where *f*_*t*_ denotes the forget gate, *i*_*t*_ refers to the input gate, *c*_*t*_ denotes the cell state, *o*_*t*_ is the output gate, *h*_*t*_ is the regular hidden state, σ indicates sigmoid function, and ◦ is the Hadamard product.

#### Bidirectional Long short-term memory networks (BiLSTM)

In the traditional recurrent neural network model and LSTM model, the propagation of information is only in forward direction. This results in computation of an output vector only based on the current input at time t and the output of the previous unit. The back propagation of information in network is achieved by merging two bidirectional recurrent neural network (BiRNN) and LSTM units, one for forward direction and one for backward direction. This helps in capturing contextual information and enhances learning ability [34].

In bidirectional LSTM, outputs of two LSTM networks are stacked together. The first LSTM is a regular sequence starting from the starting of the paragraph, while the second LSTM is a standard sequence, and the series of inputs are fed in the opposite order. The first hidden state is denoted by ht^forward^ whereas second LSTM unit’s hidden state is denoted by ht^backward^. After processing data, the final state ht^Bilstm^ is computed by concatenating the two hidden states as given in Eq. 3.

3 $${\text{Ht}}^{{{\text{bilstm}}}} = {\text{ ht}}^{{{\text{forward}}}} \oplus {\text{ht}}^{{{\text{backward}}}}$$

where ⊕ denotes a concatenation operator.

#### Convolutional neural networks (CNN)

Convolution neural networks (aka CNN), originally incorporated for image processing tasks, have become very efficacious in different NLP and text classification applications. The network identifies correlations and patterns of data via their feature maps. Information about local structure of data is extracted by applying multiple filters with different dimensions [35].

## Data preprocessing on Roman Urdu microtext

Big social media data in Roman Urdu language is inconsistent, incomplete, or precise, missing in certain behaviors or trends, and is likely to incorporate many errors. Roman Urdu users highly deviate language norms while communicating on social media. Hence data preprocessing is immensely significant. Some major data preprocessing steps applied on Roman Urdu microtext are detailed below.

### Handling Unicode and encoded text formats

Unicode scheme provides every character in natural language text a unique code from 0 to 0 × 10FFFF. The uncleaned Roman Urdu data comprised of special symbols, emojis, and other typical stray characters represented using Unicode. We used Unicode transformation type 8 encoding to convert the data. This data was converted and handled using re and string modules in python.

### Text cleaning

Text cleaning is essential step to eliminate or at least reduce noise from Microtext. This step comprised of case transformation, removal of punctuations and URLs, elimination of additional white spaces, exclusion of hashtags, digits & special character removal and removal of metadata/non-linguistic features.

### Tokenization

Tokenization is immensely essential phase of text processing. It is the process of generating tokens by splitting textual content into words, phrases, or other meaningful parts. It is generally a form of text segmentation [36]. Tokenization was performed using Keras tokenizer to prepare the text for implementing deep learning networks.

### Filtering stop words

Stop words are non-semantic division of text in natural language. The necessity that stop words should be eliminated from text is that they make the text higher dimensional with redundant features which are less significant for analysts. Removing stop words reduces the dimensionality of term space [37]. Development of domain specific stop words in Roman Urdu language automatically using statistical techniques and bilingual experts’ input, comprising of 173 words is detailed in our previous work [30]. Insignificant Roman Urdu words were typically articles such as ek (ایک), conjunctions and pronouns such as tum (تم), tumhara (تمھارا), us (اُس), wo/who (وہ), usko (اُسکو), preposition such as main (میں), pe (پے), par (پر), demonstratives such as ye (یے), inko (انکو), yahan(یہاں), and interrogatives such as kahan (کہاں), kab (کب), kisko (کس کو), kiski (کس کی) etc. Stop words were removed from Roman Urdu corpus, leaving behind the index terms which are important.

### Mapping slangs and contractions

**Fig. 2:**
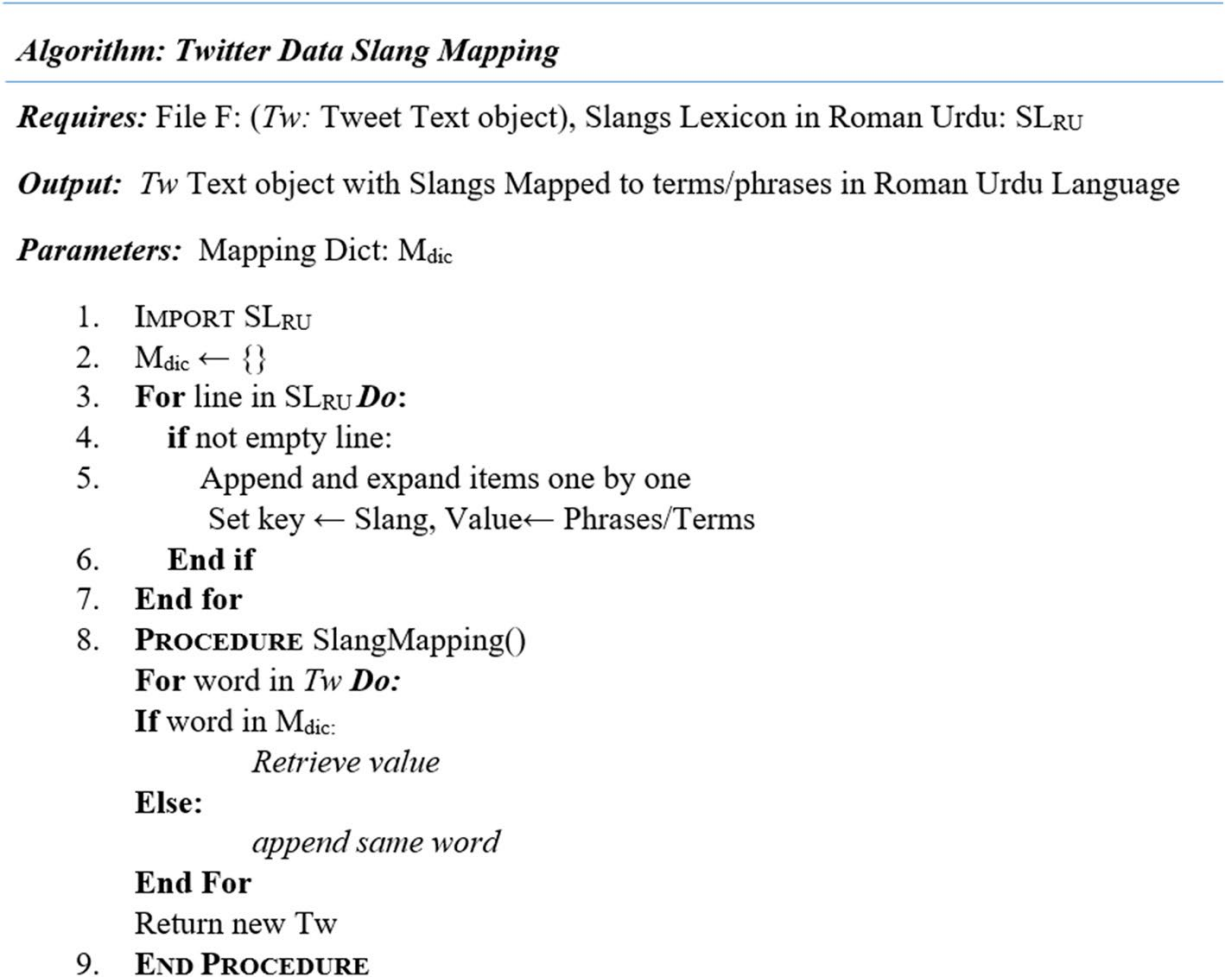
Mapping process for slangs in Roman Urdu

Existing libraries, APIs and toolkits in python language primarily support preprocessing functions for English and other mature languages. They can be partially used for Roman Urdu language. Moreover, most of the communication in Roman Urdu comprises of bully terms being used as slangs. High dimensional textual data also suppress significant features. Hence contraction mapping is mandatory for dimensionality reduction and to capture complex bullying patterns. Currently, Pycontractions Library [38] only supports English contraction mapping process. To address this problem, the study developed data slang mapping process. To map slangs to original terms and phrases in Roman Urdu language, we created Slang lexicon in Roman Urdu (SLRU) which also included Roman Urdu abuses and offensive terms used as a norm by Roman Urdu users. SLRU is in the form of a dictionary. It comprises of the key: value pairs, where key is the slang and value is its equivalent Roman Urdu phrase/term such as “AFIK”: “Jahan tak mujhay pata hai”, ASAP: “Jitna jaldi ho sakay”, “tbh”: “Sach main” and so on. The process of slang mapping is detailed in Fig. 2.

**Fig. 3:**
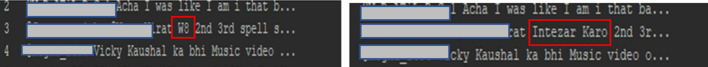
Mapping on Roman Urdu Data

The results of mapping process are highlighted in Fig. 3.

## Experimental setup

This section discusses implementation of proposed neural network architecture and all hyper-parameter settings. All the experiments were performed on 11 Gen, core i7, 4 cores, 8 logical microprocessors, with 2.8 GHz processor speed, 256 GB Solid State Drive and python version 3.8, 64 bits.

### Proposed model implementation and hyper-parameter settings

All models were implemented and trained in Keras; a high-level neural network API that works with open-source machine learning framework called TensorFlow [39]. All the implementation was accomplished using PyCharm. The optimal parameters and results were achieved through repeated experimentations.

Data was split into training and testing datasets. The data split was 0.8 for training and 0.2 for testing i-e 80% of data instances were used for training and 20% were holdout for testing and validation purpose. The sets were made using shuffled array. This allows model to learn over different data instances. Moreover, it helps to uncover reliability of model and consistency of results over repeated executions. Random state is also generated using numpy.random [40] for random sampling during splitting of data to ensure reproducible splits.

Textual input data must be integer encoded. In RNN-LSTM architecture, a sequential model was created. Initially an Embedding layer was added to the network and textual Roman Urdu data was provided as an input. Embedding layer embedded high dimensional text data in low dimensional vector space for generating dense vector representation of data. Embedding was formed using 2000 features and 128 embed dimensions. The experiment was initially executed on 20 epochs and 50 batch size. The batch size was based on the fact that model was having single lstm layer, and comparatively took lower training and validation time per step. The execution time for each epoch was approximately 10 ms. SpatialDropout1D was used with rate of 0.3. It helped to regularize the activations and maintain effective learning rate of the model. For updating network weights iteratively, this work uses binary cross entropy loss function and Adam optimizer. Sigmoid activation function was also implemented. It is denoted by f(x) and is defined as:

4 $${\text{f }}\left( {\text{x}} \right) \, = {1}/\left( {{1 } + {\text{ e}}^{{( - {\text{x}})}} } \right)$$

The Spatial Dropout layer was implemented instead of a simple Dropout. The major reason being was to retain the context of textual data established by neighboring words. Dropping random words (except for stop words, which were already removed during preprocessing step) can highly affect the context of uttered sentences and ultimately the performance of model. We incorporated two hidden dense layers denoted by D_1_ and D_2_. The output of each hidden layer was computed to get the final output for cyberbullying text detection.

Keras tokenizer was used to accomplish pre-tokenization of all the data required for the implementation of RNN-biLSTM model. We created a sequential model with Embedding layer having 2000 maximum features. Subsequently a biLSTM layer comprising of two LSTM layers, one to read sequence in forward direction and other in backward direction, each with 64 units was added. Hidden layer (H_1_) was formed using sigmoid activation function. For down sampling the feature maps, Dropout layer was added with 0.2 dropout rate. Moreover, we used 128 batch size to utilize low to moderate computational resources while still not slowing down the training process. Batch size highlights number of samples processed by model before updating of internal parameters. To combat overfitting, we added second dropout layer with rate of 0.25. Adam optimization was used with learning rate of 0.01 since batch size was not too small. For this model, we used binary cross entropy loss function. As the Epochs increase, the generalization ability of the model improves. However, too many epochs also lead to the problem of overfitting. The model was executed over different number of Epochs and average execution time for each epoch was 13 to 15 ms. The performance of model stabilized over 20 Epochs, above which the improvement was almost negligible.

In CNN model, initially the sentence was transformed into matrix where each row of matrix represented word vectors representation of data. We used 1000 features and 32 dimensions. Two convolutional filters were applied with 8 and 16 filters and 3 kernel size. Each filter was used to perform one dimensional convolution on word embeddings. Both Layers were 1D in nature. We set two dropout layers with dropout rate of 0.25 to improve generalization ability of developed model. Hidden layers with Relu and sigmoid activation functions were used. To extract most salient and prominent features, global maximum pooling layers were used with pool size = 2. Flatten layer was created after convolutional layers to flatten the output of the previous layer to a single long feature vector. The experiment was simulated over different Epochs. However, results got stable at 30 epochs.

## Results and discussion

Empirical evaluation of cyberbullying detection scheme performance in Roman Urdu and experimental setups is accomplished via accuracy, precision, recall, and f1 measure metrics.

**Fig. 4:**
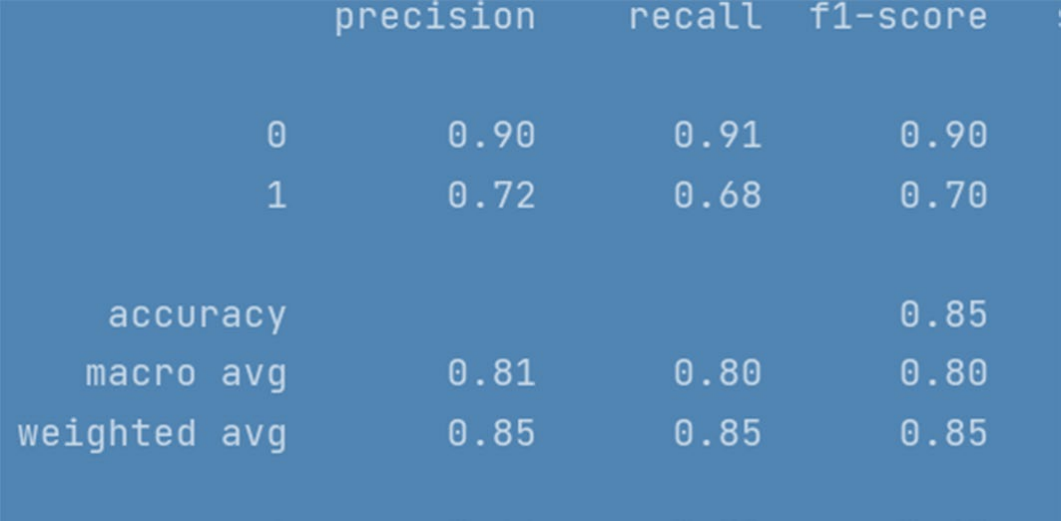
RNN-LSTM evaluation Results

All the implemented models were executed several times over number of epochs to get consistency in evaluation parameters until it was a minor difference of ± 0.1. The results for LSTM are depicted in Fig. 4. To ensure results validity and reliability, for a comparatively less skewed dataset, F1 measure (i-e a harmonic mean of precision and recall) is used as an evaluation metric. Furthermore, we have also reported macro and weighted average scores across all the classes. The evaluation results of RNN-LSTM are given in Fig. 4.

**Fig. 5:**
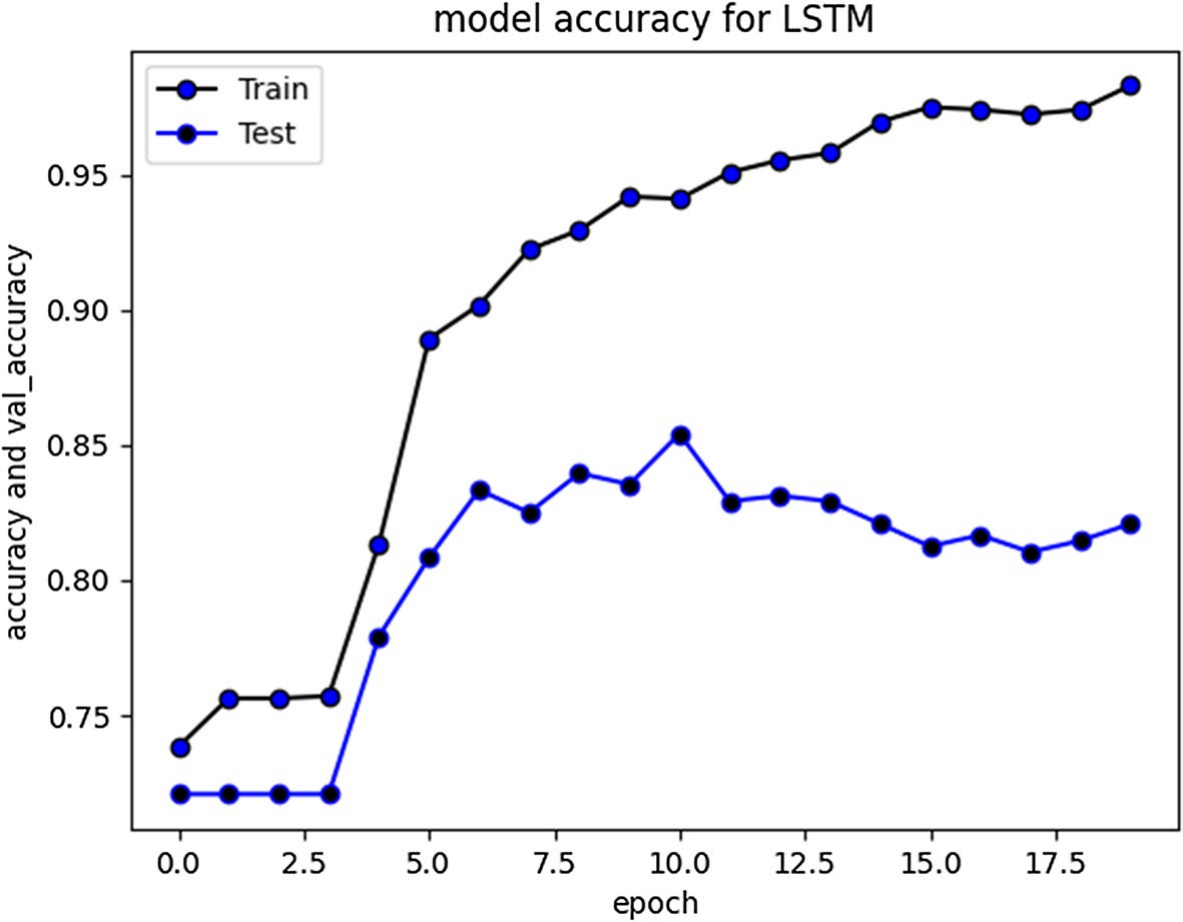
RNN-LSTM Model accuracy graph for 20 epochs

**Fig. 6:**
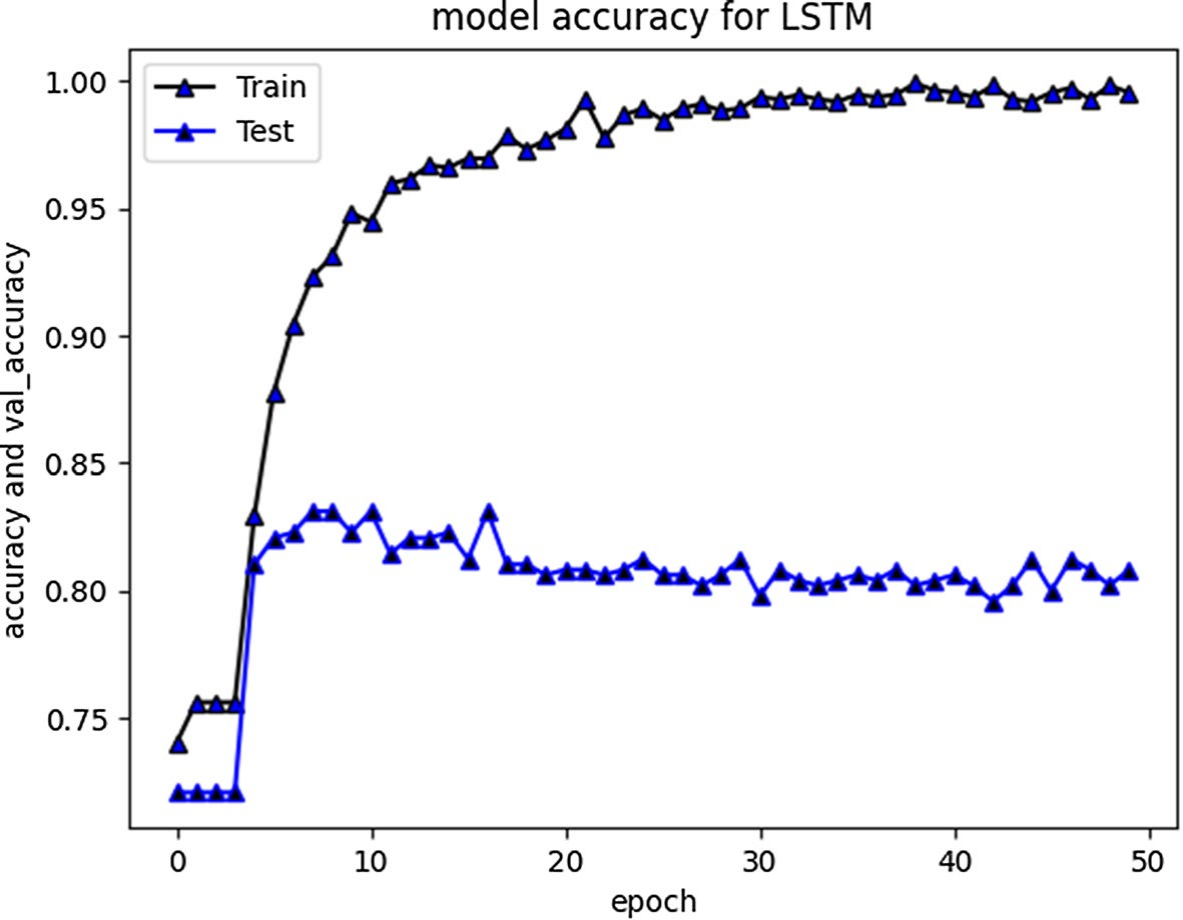
RNN-LSTM Model accuracy graph for 50 epochs

F1 score for RNN-LSTM over cyberbullying class was only 70%, however for non-cyberbullying class, score was 90%. We observed that nearly all the instances of majority class of non-cyber bullying are correctly classified by this model. The experimental simulation depicting model accuracy and validation accuracy during training and validation phases, before and after stabilization of evaluation parameters is represented in Figs. 5 and 6 respectively.

**Fig. 7:**
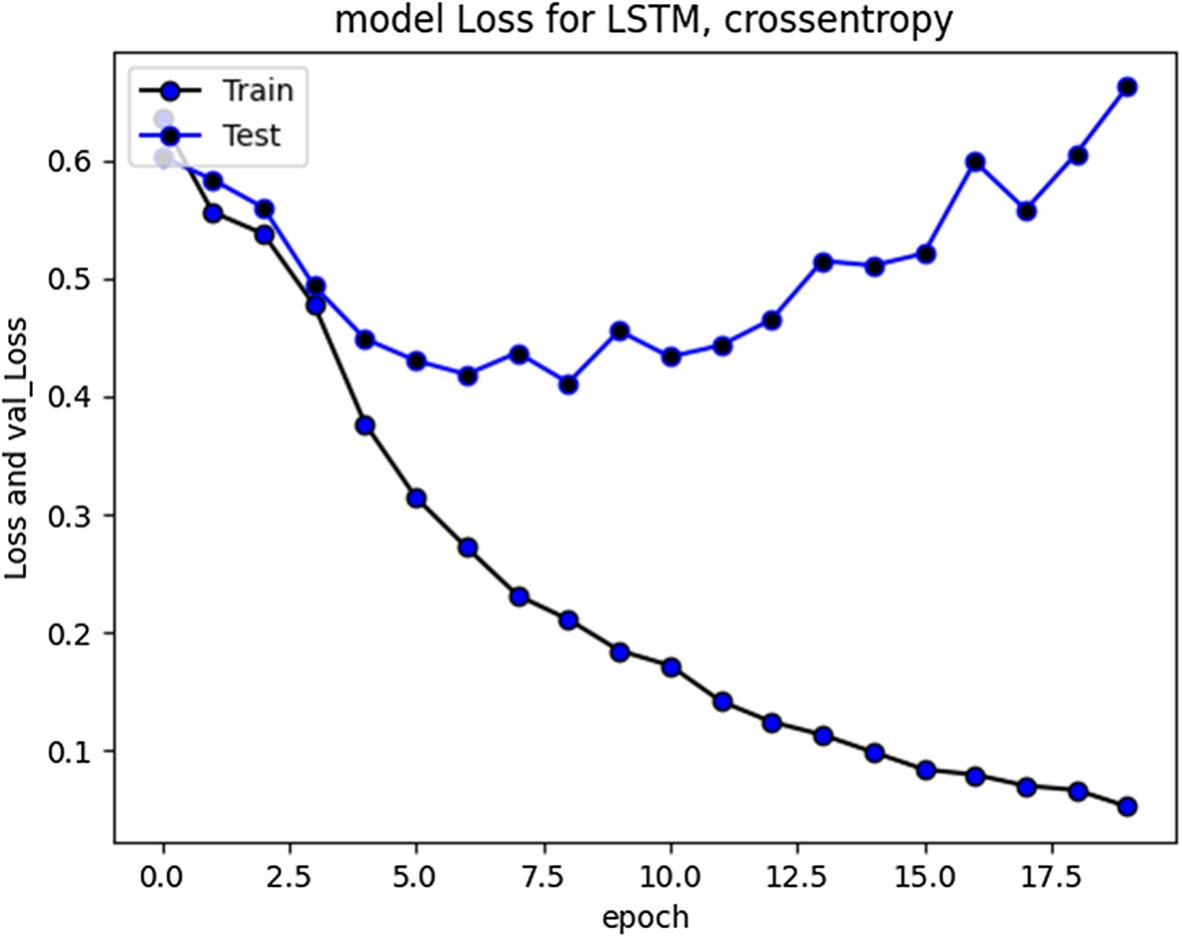
RNN-LSTM Model loss plot- Binary Cross entropy for 20 epochs

**Fig. 8:**
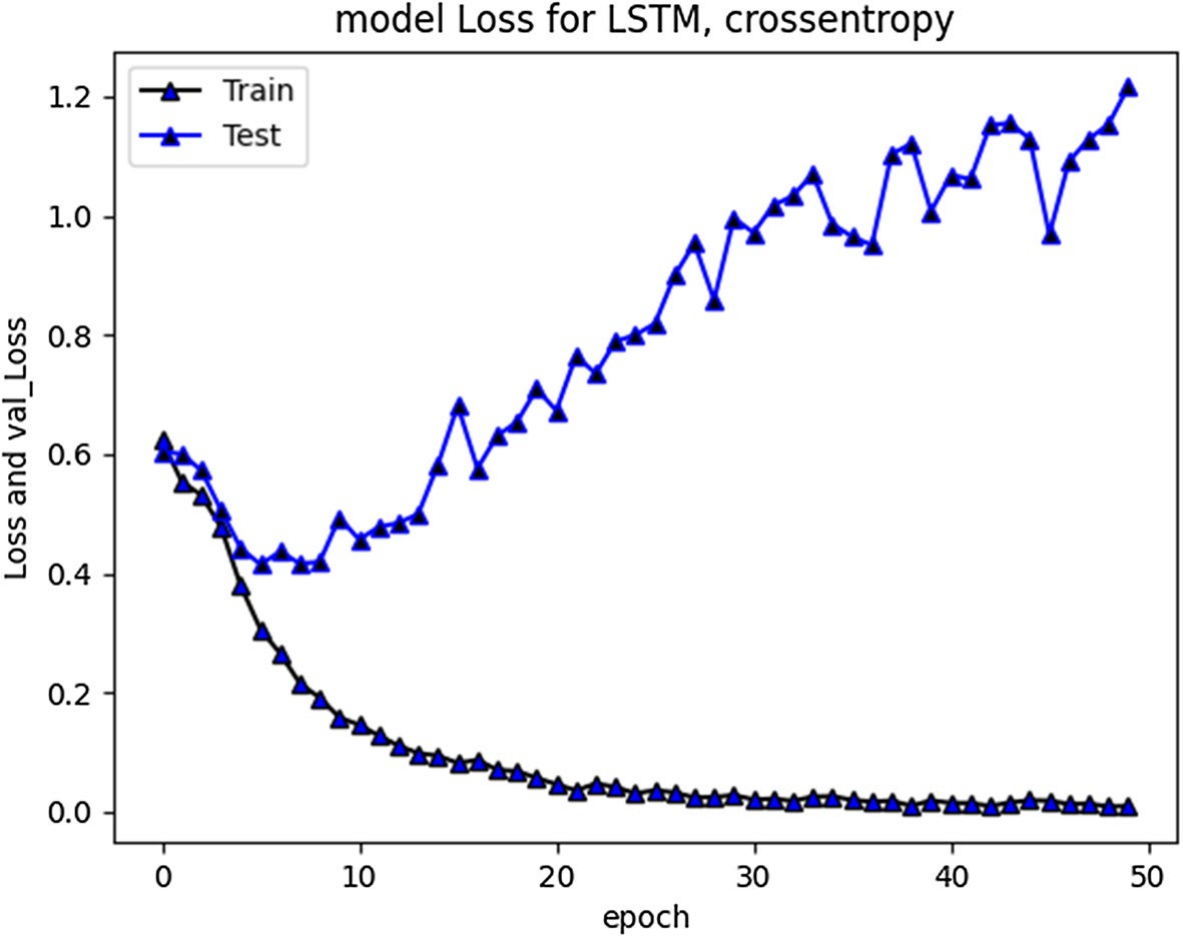
RNN-LSTM Model loss plot- Binary Cross entropy for 50 epochs

The accuracy improved over subsequent epochs. However, after 20 epochs it got stabilized. The average accuracy produced by this model was 93.5% during training and 85.5% during validation. Overall curve variation is indicating that no overfitting problem arise. The model loss during training and validation loss during testing over 20 and 50 epochs is shown in Figs. 7 and 8 respectively. The cross-entropy loss considered during configuration over different epochs converged well, thus indicating optimal model performance.

**Fig. 9:**
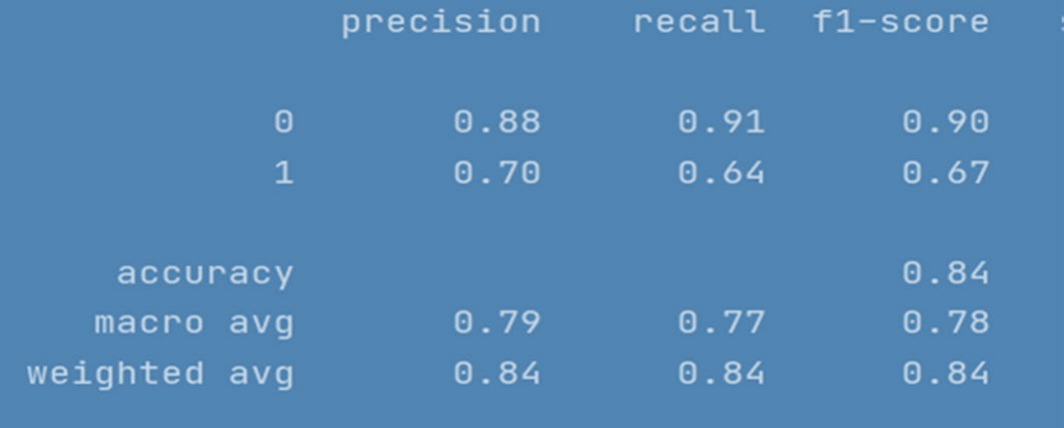
RNN-BiLSTM evaluation Results

The evaluation results of RNN-BILSTM model over 20 epochs are given in Fig. 9.

**Fig. 10:**
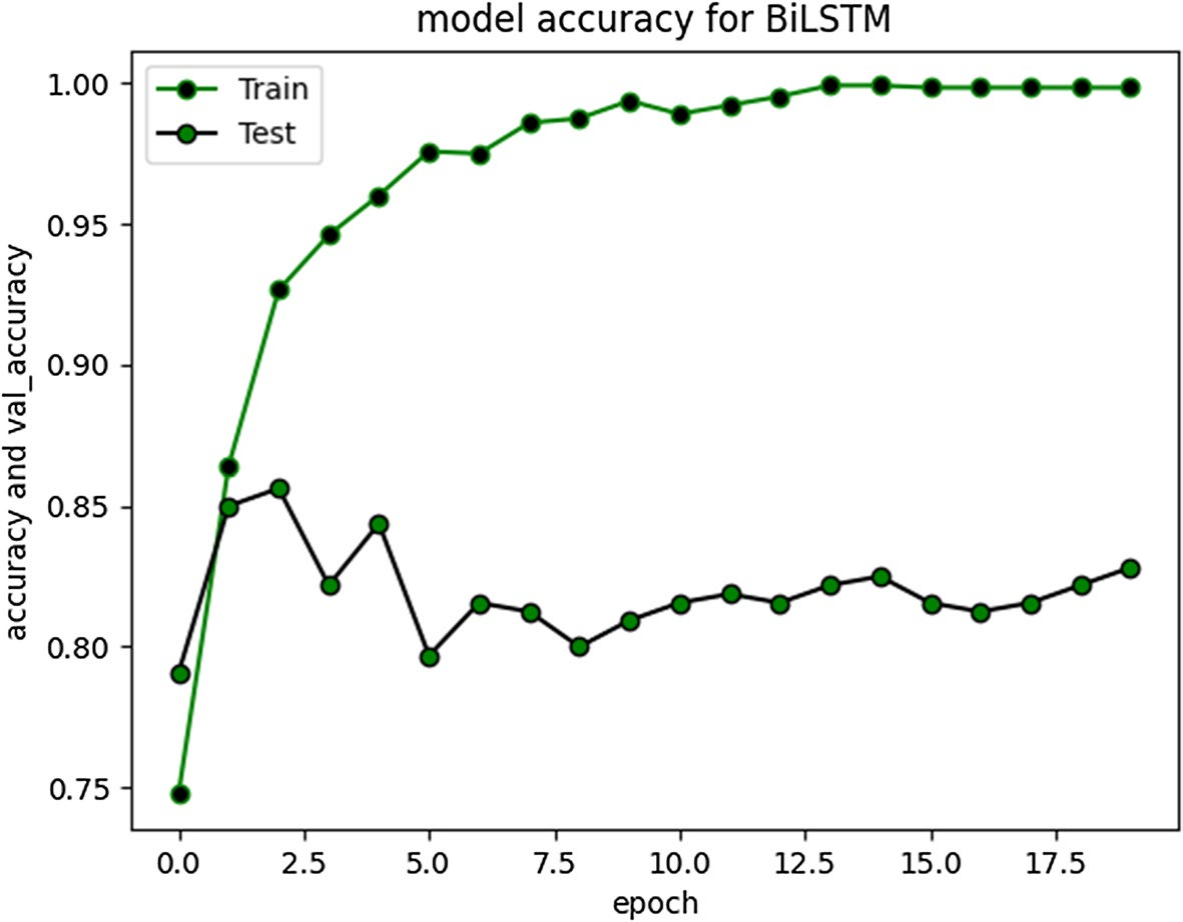
RNN-biLSTM Model accuracy plot for 20 epochs

**Fig. 11:**
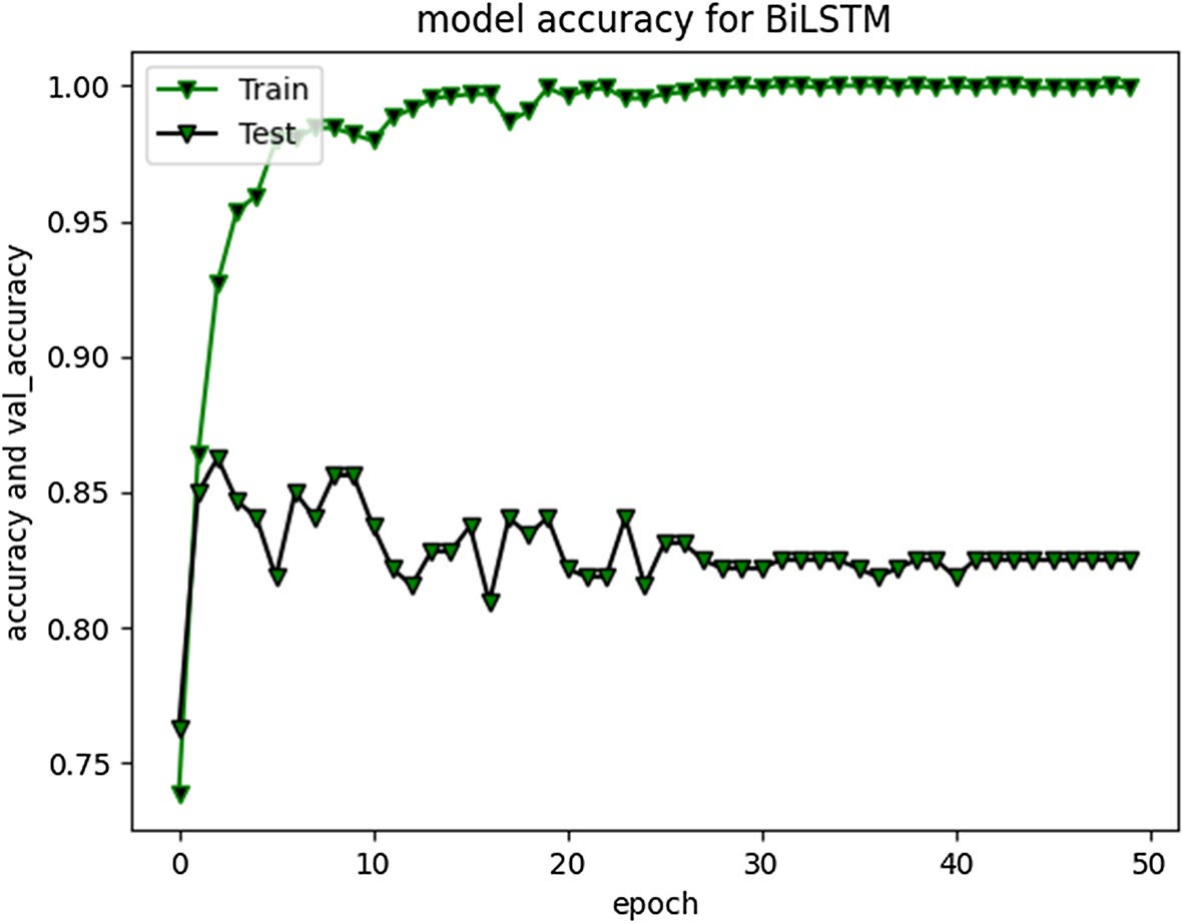
RNN-biLSTM Model accuracy plot for 50 epochs

RNN-LSTM also performed reasonably well for cyberbullying detection task on Roman Urdu data. F1 score for non-cyberbullying content prediction was 90% whereas for cyberbullying content, the score was 67% only. This indicates that model erroneously classified/misclassified some of the aggressive class instances and TN rate was at average. Figs. 10 and 11 are depicting model accuracy and validation accuracy for RNN-BiLSTM.

**Fig. 12:**
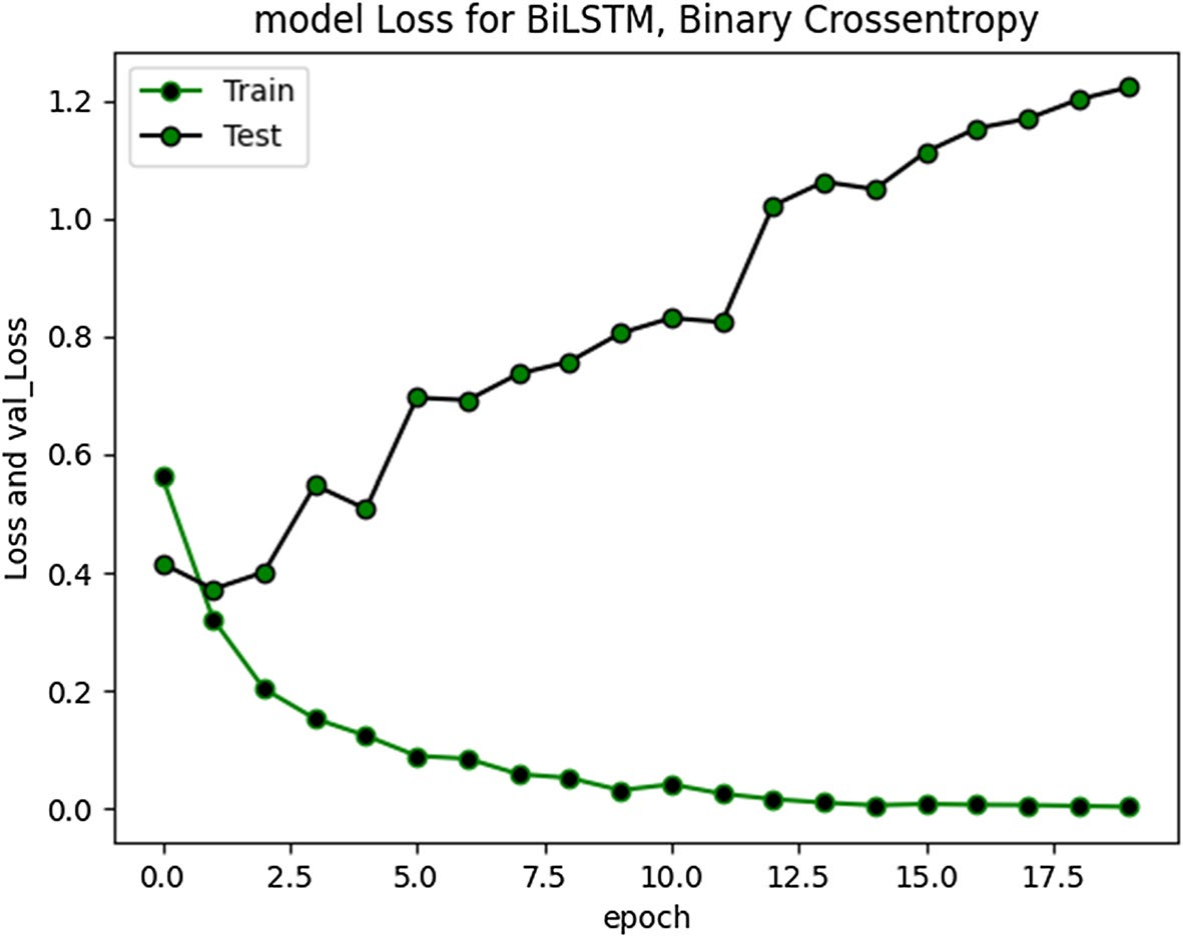
RNN-BiLSTM Model loss plot- Binary Cross entropy for 20 epochs

**Fig. 13:**
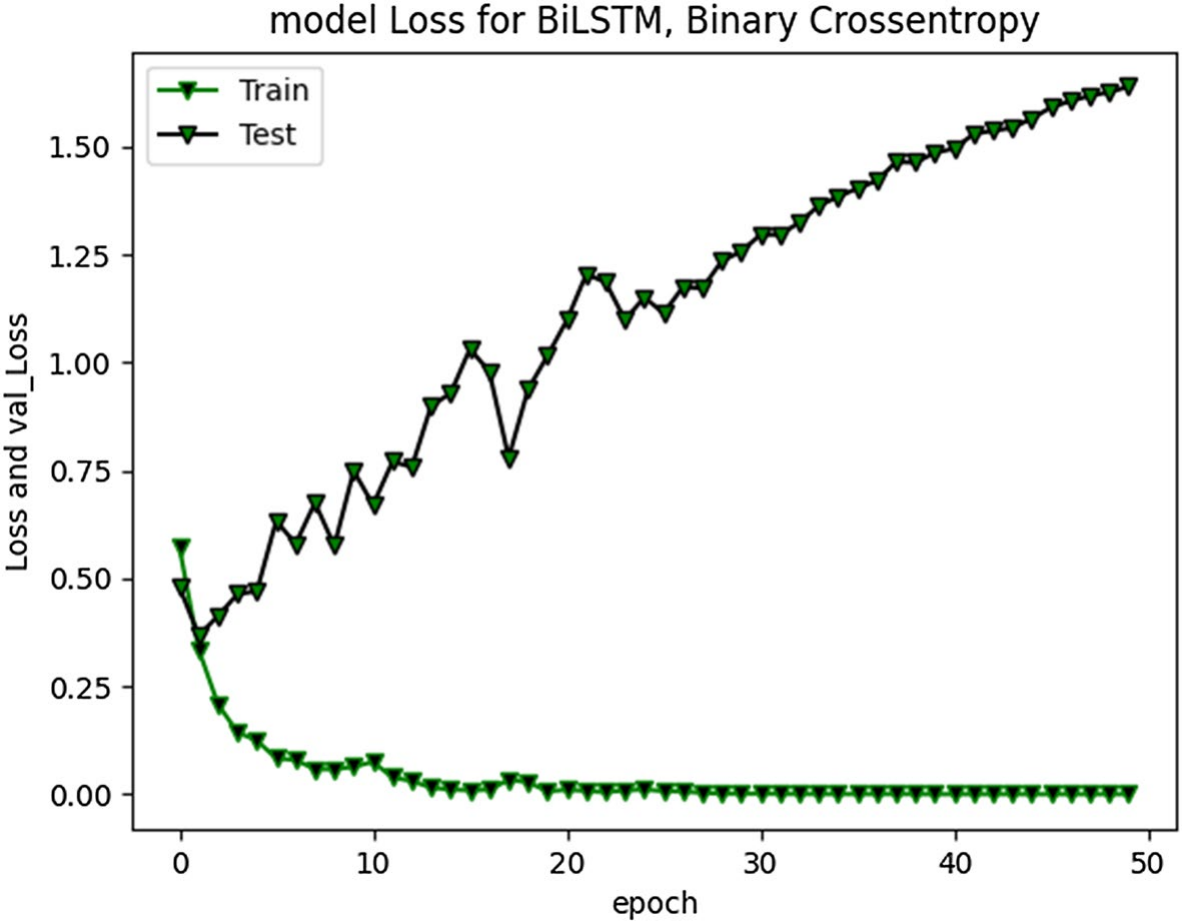
RNN-BiLSTM Model loss plot- Binary Cross entropy for 50 epochs

The accuracy improved highly during training process up to 20 epochs. Overall average accuracy was 97% in training and 85% on validation set. 20% of the data was used for as a validation set, as stated earlier. During experimentation, we identified that accuracy of our model is not improving after a specific point i-e after 20 Epochs. The trivial variations can be clearly visualized from the graph in Fig. 9. Model loss and validation loss during training and testing process for RNN-BiLSTM over 20 and 50 epochs is given in Figs. 12 and 13 respectively.

The cross-entropy loss was minimal (approximately 1.2), indicating good prediction capability of developed model.

**Fig. 14:**
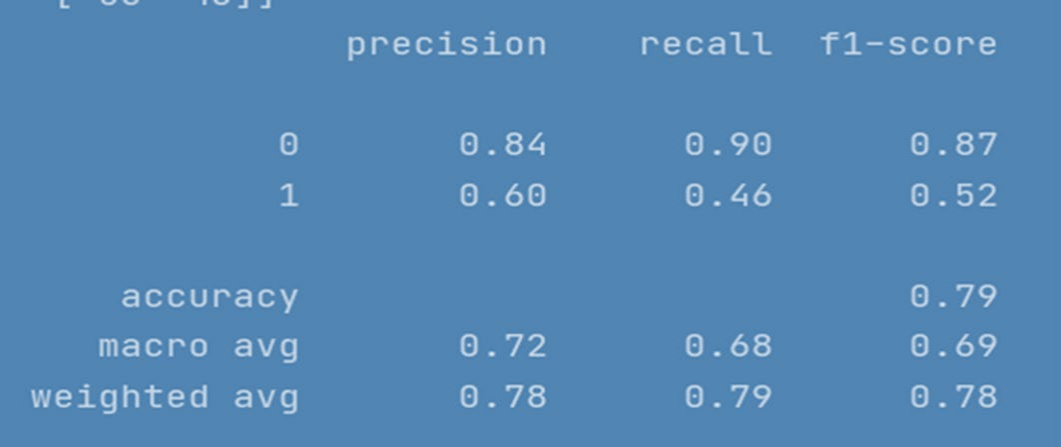
CNN model evaluation Results

Figure 14 represents the evaluation results for CNN model.

**Fig. 15:**
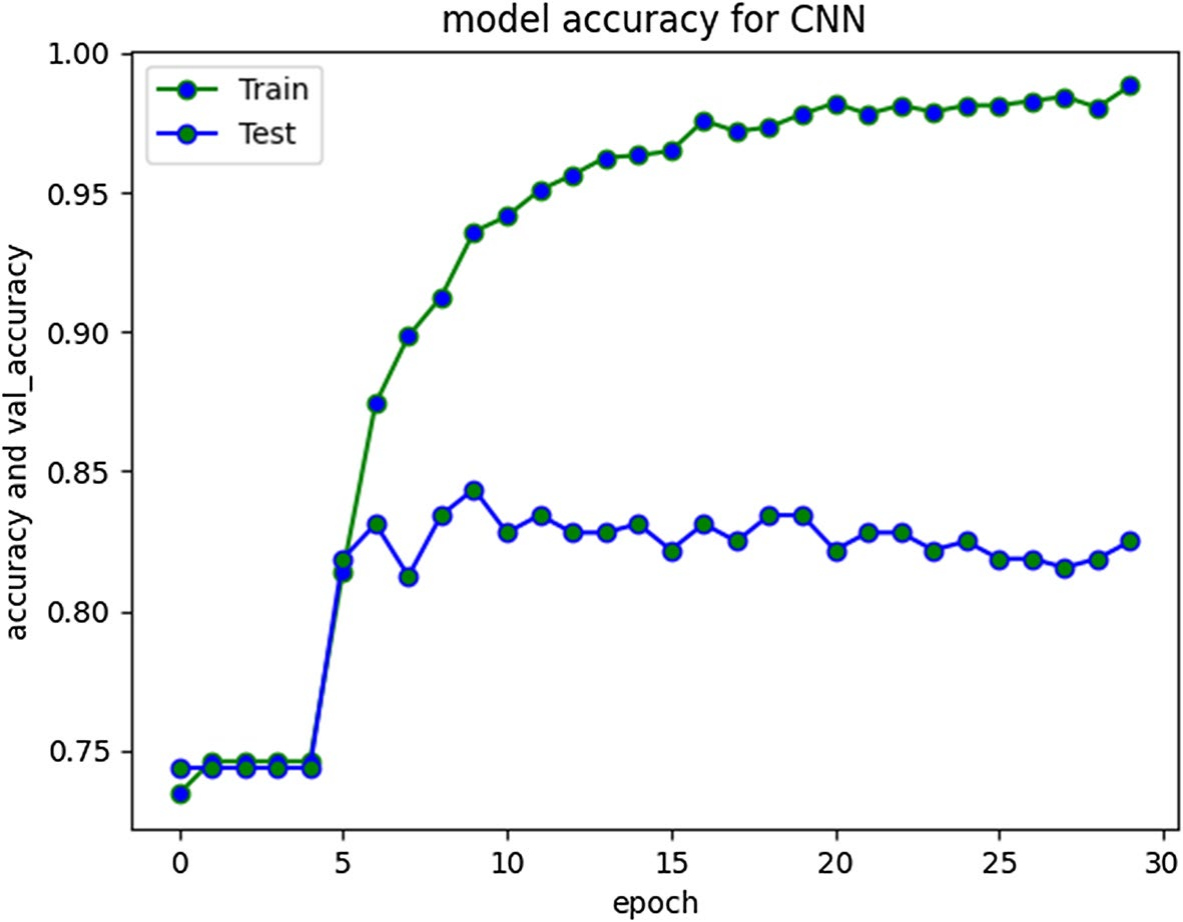
CNN Model accuracy plot for 30 epochs

**Fig. 16:**
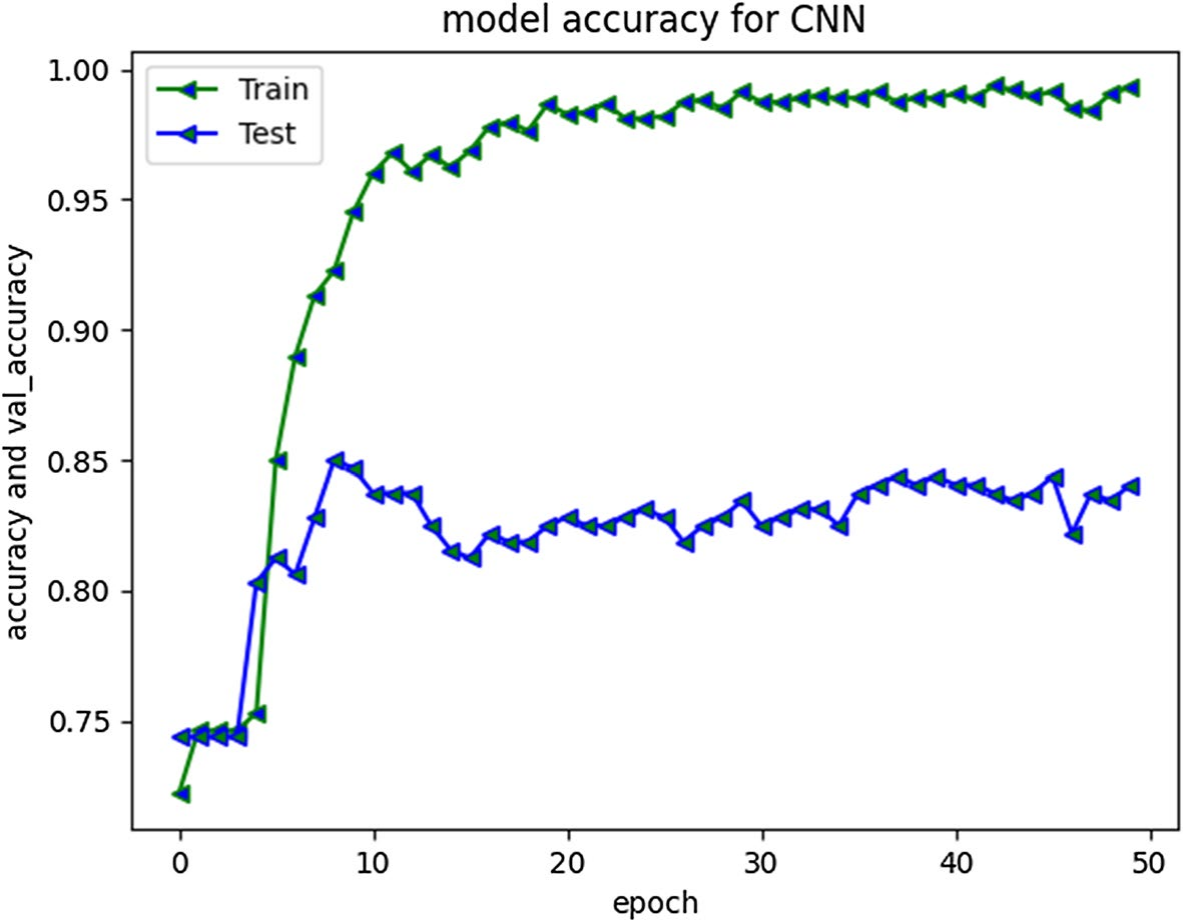
CNN Model accuracy plot for 50 epochs

CNN performed well for prediction of non-cyberbullying content, providing F1 score of 87%. However, model did not yield good efficiency for categorizing cyberbullying class, producing f1-score of 52%. The repeated experiments performed for CNN showed continuous improvements up to 30 Epochs. Figure 15 depicts model accuracy and validation accuracy. The experimental simulation over 50 epochs only shown minor improvements as represented in Fig. 16. The average execution time for Epoch was 9 ms each. The training accuracy of 98% was achieved over different executions whereas model produced 85% validation accuracy.

**Fig. 17:**
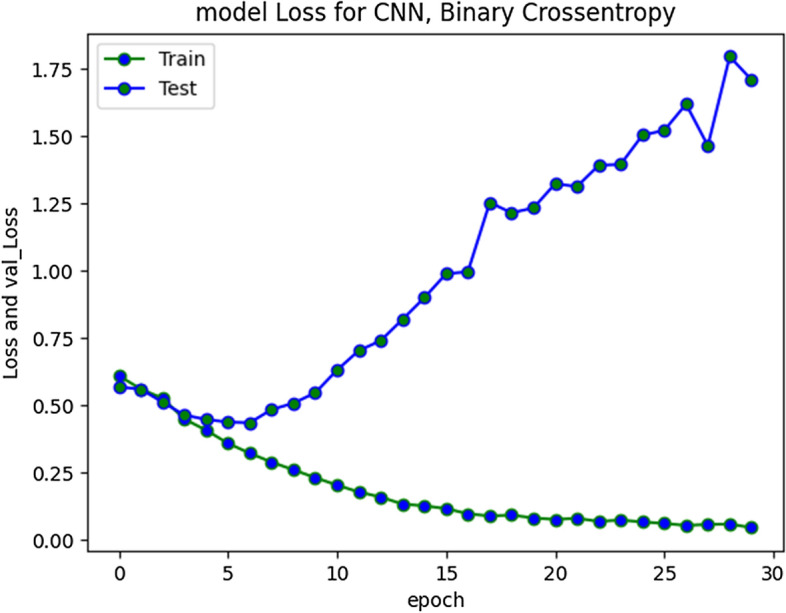
CNN Model loss plot- Binary Cross entropy for 30 epochs

**Fig. 18:**
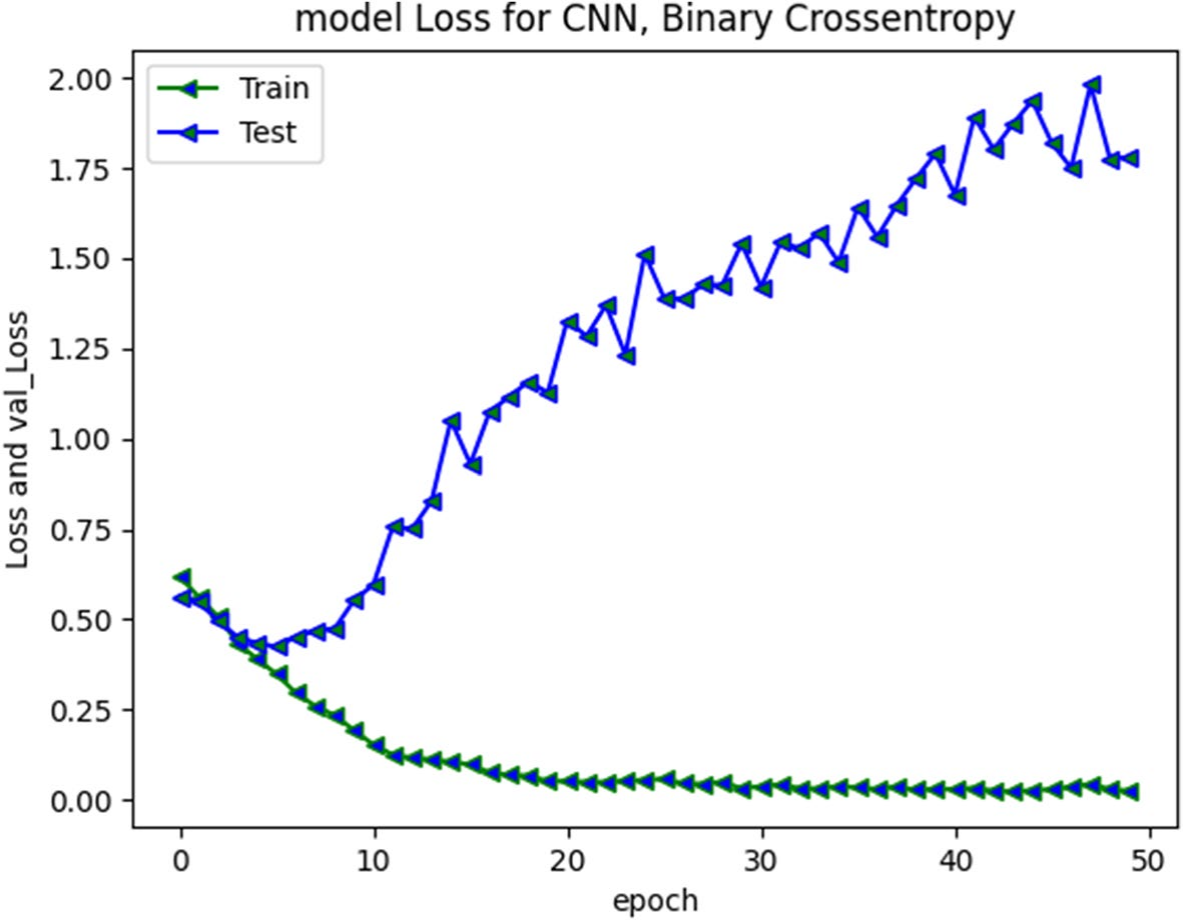
CNN Model loss plot- Binary Cross entropy for 50 epochs

CNN model loss and validation loss results at 30 and 50 epochs are presented in Fig. 17 and 18 respectively. The loss was minimal during training and converged. During validation the loss increased and diverged indicating only moderate performance over unseen instances typically from aggressive class.

The compiled model results indicating evaluation measures at stabilized epochs are depicted in Table 2.

**Table 2:** Evaluation Measures for Implemented Models

Evaluation Measures	Approach/Model					
	RNN-LSTM		RNN-biLSTM		CNN	
	0	1	0	1	0	1
Precision	0.90	0.72	0.88	0.70	0.84	0.60
Recall	0.91	0.68	0.91	0.64	0.90	0.46
F1-Score	0.90	0.70	0.90	0.67	0.87	0.52
Accuracy	0.85		0.84		0.79	
Macro average	0.80		0.78		0.69	
Weighted Average	0.85		0.84		0.78	
Approximate Loss	0.6		1.2		1.7	

## Conclusion

Cyberbullying has become an alarming social threat for today’s youth and has recently gained huge attention from research community. This research has addressed the problem of cyberbullying detection in Roman Urdu Language. Since Roman Urdu is highly resource deficient language, having different writing patterns, word structures, and irregularities thus making this work a challenging task. In this work we have presented advanced preprocessing techniques mainly a slang mapping mechanism, domain specific stop word removal, handling encoded formats and formulation of deep learning architecture to detect cyberbullying patterns in Roman Urdu language. We created experiments with vast parameters to build optimal classifier for cyberbullying tweets. The results highlighted that RNN-LSTM and RNN-BiLSTM with concatenation of forward and backward units provided better performance in 20 Epochs as compared to CNN. The existing work can be extended in numerous ways. The future studies can focus on development of ensemble models to uncover harassing and hate speech patterns. Moreover, the incorporation of context-specific features and handling of morphological variations might produce better results.

## Data Availability

The used raw dataset in this research is not publicly available. The data that support the findings of this research work are available from the corresponding author, on valid request due to privacy and ethical restrictions.

## Abbreviations

RNN: Recurrent neural network
LSTM: Long-short term memory
BiLSTM: Bidirectional long-short term memory
CNN: Convolutional neural network
SN: Social networking
RTL: Right to left
LTR: Left to right
TN: True negative
